# Community solar salt production in Goa, India

**DOI:** 10.1186/2046-9063-8-30

**Published:** 2012-12-01

**Authors:** Kabilan Mani, Bhakti B Salgaonkar, Deepthi Das, Judith M Bragança

**Affiliations:** 1 Department of Biological Sciences, BITS PILANI, K K Birla Goa Campus, Zuarinagar, Goa, 403 726, India

**Keywords:** Salt pan, Goa, Estuary, Community, *Khazan*, Tidal influx, India, Salt production

## Abstract

Traditional salt farming in Goa, India has been practised for the past 1,500 years by a few communities. Goa’s riverine estuaries, easy access to sea water and favourable climatic conditions makes salt production attractive during summer. Salt produced through this natural evaporation process also played an important role in the economy of Goa even during the Portuguese rule as salt was the chief export commodity. In the past there were 36 villages involved in salt production, which is now reduced to 9. Low income, lack of skilled labour, competition from industrially produced salt, losses incurred on the yearly damage of embankments are the major reasons responsible for the reduction in the number of salt pans.

Salt pans (*Mithagar or Mithache agor*) form a part of the reclaimed waterlogged *khazan* lands, which are also utilised for aquaculture, pisciculture and agriculture. Salt pans in Goa experience three phases namely, the ceased phase during monsoon period of June to October, preparatory phase from December to January, and salt harvesting phase, from February to June. After the monsoons, the salt pans are prepared manually for salt production. During high tide, an influx of sea water occurs, which enters the reservoir pans through sluice gates. The sea water after 1–2 days on attaining a salinity of approximately 5ºBé, is released into the evaporator pans and kept till it attains a salinity of 23 - 25ºBé. The brine is then released to crystallizer pans, where the salt crystallises out 25 - 27ºBé and is then harvested.

Salt pans form a unique ecosystem where succession of different organisms with varying environmental conditions occurs. Organisms ranging from bacteria, archaea to fungi, algae, etc., are known to colonise salt pans and may influence the quality of salt produced.

The aim of this review is to describe salt farming in Goa’s history, importance of salt production as a community activity, traditional method of salt production and the biota associated with salt pans.

## Background

Goa, together with Daman and Diu, was a province under the Portuguese rule from 1510 and referred to as *Estado da India*. Goa was annexed by India on 19th December 1961 and liberated from the Portuguese rule [1]. Solar salt production in Goa had been an important activity through its history.

Goa experiences a tropical monsoon climate with hot summers followed by long monsoons from June to October. Goa has 9 rivers, most of them forming estuaries, the major being river Mandovi and Zuari. These rivers experience high tidal influx during summers and therefore the salinity varies during monsoon (2–3ºBé) and non-monsoon times (4–5ºBé) [2]. Various factors such as favourable climatic conditions and easy accessibility to sea water have aided salt production through natural evaporation in Goa.

Today, solar salt production has become a declining industry due to low income generated, competition from industrially produced iodized salt, yearly damage and repairs of the embankments and pollution. Currently there are 9 villages producing salt each having a few operational salt pans.

### Historical background

Solar salt production in Goa, described as a traditional village industry, has been practiced for the past 1,500 years by various communities [3,4]. Since most of the rivers form estuaries and experience tidal influxes, salt production was started mainly in the coastal villages. Salt served as an important trade commodity too, playing an important role in the economy of Goa. The salt produced in the Goan salt pans was considered to be of superior quality and was exported to Burma, Thailand and other Asian countries [5]. With the Portuguese colonization of Goa in 1510, the salt production gained a huge momentum because of the increased demand for consumption. Portuguese cuisine required surplus salt and it was also used in balancing the hull of ships on sailing. With the maritime dominance of Portuguese, salt produced in Goa was exported even to the Middle Eastern countries. Salt was thus the major export commodity of the ‘*Estado da India*’ through the Mormugao port [6-11].

Due to the 1878 Anglo – Portuguese Treaty, the British monopolised the salt production in Goa and resold it to the Portuguese. After the British took over the salt pans, the average quota of salt for an individual was reduced from 14.5 kg to 6.5 kg. This had forced many people to reduce the intake of salt which in turn resulted in hyponatremia [12]. With the annexion of Goa by India in 1961, the salt production through natural evaporation faced severe decline, which continues till date. Competition from iodized salt, availability of salt at less cost from other states and lack of skilled labour forced the salt pan operators to abandon the salt pans and look for other employment opportunities [13].

### Areas where salt is produced

**Figure 1:**
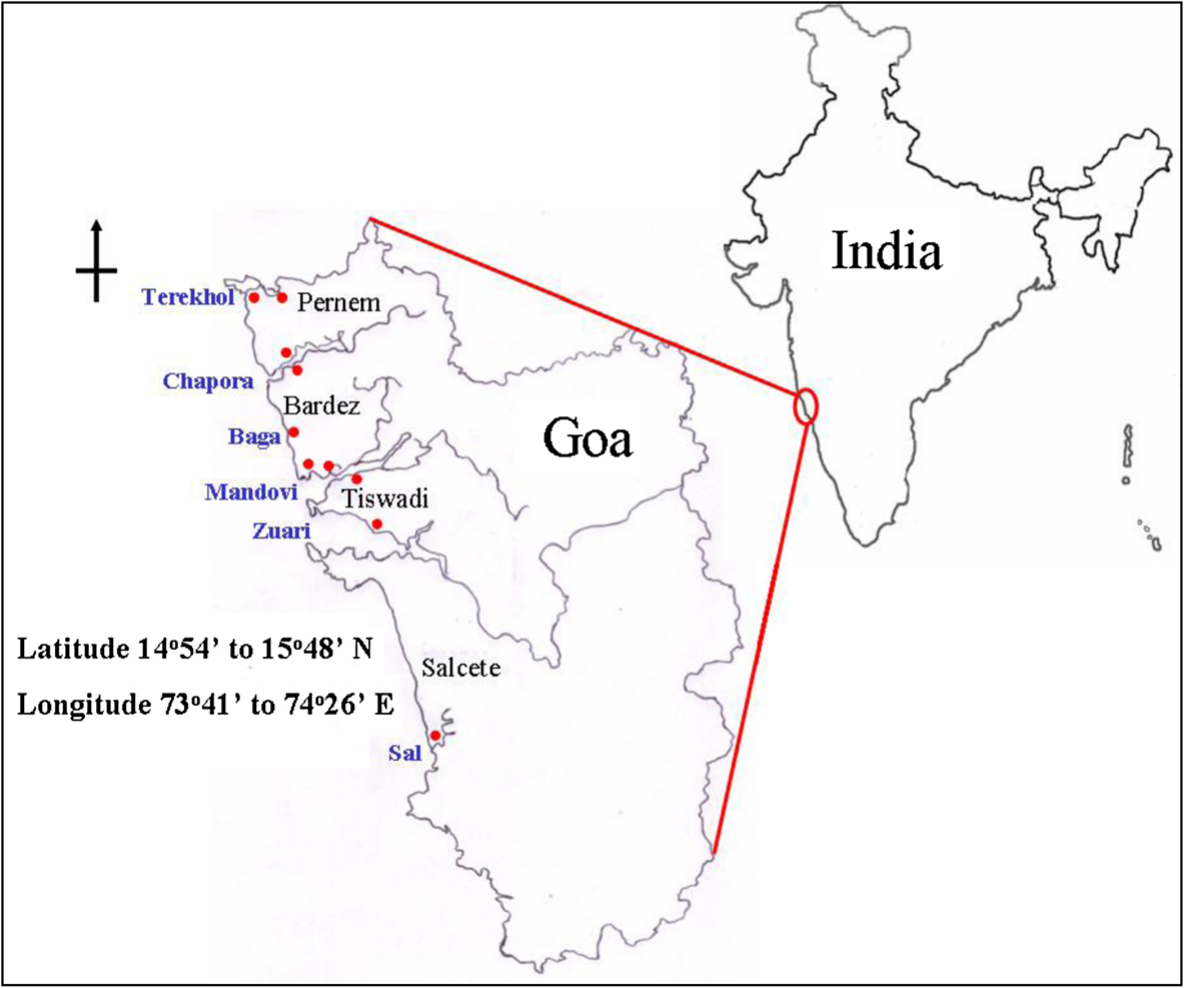
Map of Goa showing the four salt producing talukas (Pernem, Bardez, Tiswadi and Salcete) of Goa (located on the west coast of India) and their location on different river estuaries (Terekhol, Chapora, Baga, Mandovi, Zuari and Sal).

**Figure 2:**
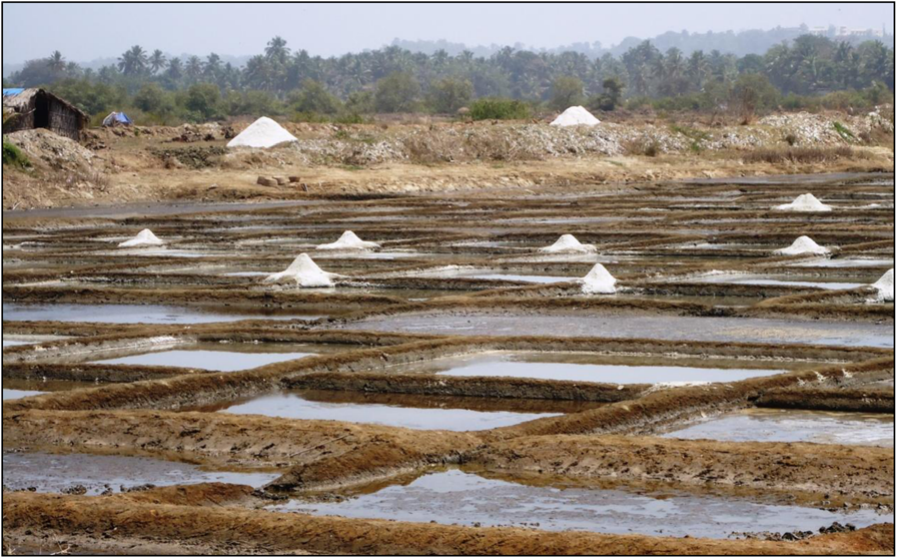
**Salt production at Siridao located in Tiswadi taluka of Goa.** Precipitated salt is heaped and kept for drying at the corners of the crystallizer pans.

Goa has an area of 3,702 sq. km and lies between 14^o^54^′^ to 15^o^48^′^ North and 73^o^41^′^ to 74^o^26^′^ East, with a coastline of about 110 km. The Arabian Sea borders Goa on the west, Maharashtra to the north and Karnataka to the east and south. Goa consists of 443 villages with a population of 1.2 million. A former union territory, Goa was added as the 25*th* state of Indian union on 30*th* May, 1987. It is divided into 2 districts, North Goa and South Goa together consisting of 12 talukas [14]. Goa receives an annual rainfall of about 280 to 480 cm, most of it during the months of June to October. The salterns receive high intensity sunlight and strong winds, making salt production a successful activity in Goa only during the summer. Salt production was concentrated around in 36 villages mainly in the four talukas Pernem, Bardez, Tiswadi and Salcete. These villages lie on the estuaries of the Terekhol, Chapora, Baga, Mandovi, Zuari and Sal rivers (Figures 1 and 2). Currently, the number of salt producing villages has drastically reduced to 9 and the total area under current salt production is about 2,978 ha [15]. Because of the landscape and ownership of lands, all the salt pans in Goa come under the category of a small scale production, which is less than 4.04 ha and, owned by the private sector. In 1876, Goa’s salt production was about 44 kton and in 1961, it reduced to 31 kton. In 2011, Goa’s total salt production was a mere 2.1 kton. This is very low when compared with India’s total salt production in 2011–2012, which was about 22179 kton [16].

India is the third largest salt producer only after China and USA. At the time of independence, India’s annual salt production was 1900 kton making it an importer from United Kingdom and Adens. But within a short span of time, India filled the gap in supply chain and became an exporter of salt. The main contribution comes from the states of Gujarat, Rajasthan and Tamil Nadu, which is about 90% of the country’s total production [16]. Salt production methods vary widely among the different salt producing states. Apart from sea water, sub-soil brine, lake brine and rock salt deposits are also being used for salt production [17]. Private sector plays a major role in salt production, contributing 90.3%. Most of the salt pans have iodisation plants located nearby for fortifying the salt with iodine and iron. Looking at the scenario of other Indian salt producing states, Goa’s salt production fails to meet its local demand.

### Communities involved in salt production

A unique organizational structure called *comunidade*, headed by a hereditary descendant, involves in governing villages and regulating the agricultural activities in Goa. Each village constitutes a *comunidade* and has its own rules depending on the local customs. This is one of the oldest administrative setup, which is in existence for the past thousand years and has been recognized by the constitution. In the past, *comunidade* was responsible for reclaiming the waterlogged lands (*khazans*) along the coasts and making them suitable for agricultural activities, aquaculture, pisciculture and salt production. Therefore all the activities in these *khazan* lands were regulated by *comunidade* including salt production. The income obtained from these *khazan* lands were utilized for the community development activities [18].

Five communities are involved in the salt production. They are *Mithgaudas, Gauddos, Bhandaris, Agris* and *Agers*[4,19]. They either own the salterns or are employed by one of these communities. The salt making art was pioneered by the ancestors of the *mithgauda* community known as ‘*Shamans*’ [20]. The *mithgauda* is a subdivision of the *gauda / govada* community mainly settled in Corgaon and Agarwada region of Pernem taluka. They are believed to have migrated from the konkan belt of Maharashtra [19]. Even though the procedure for producing salt followed by all the communities is the same; with some minor variations such as the collection of salt crystals from crystallizer pans. This indicates the evolution of salt production process within a community over a period of time. In the past salt production alone was the only source of income for the people of these communities. With the increase in profits and the importance emphasised by Portuguese on salt production, many people who had lands accessible to seawater started salt production. Salt pans also provided employment opportunities for the migrant workers from the neighbouring states. [4,20].

### Process of salt production

*Khazan* lands of Goa are reclaimed mesohaline agricultural lands in the estuarine regions. At most of the places these *khazan* lands are surrounded by a thick lush of mangrove vegetation. The salinity and tidal influx is regulated by embankments (dykes/bunds/*mero*) and sluice gates (*manos*) [21]. These sluice gates are symbols of rich cultural heritage and engineering skills. *Khazans* are described as contour controlled, topo-hydro engineering, agro-economic and agro-ecological sustainable productive systems. These *khazan* lands are utilized for agriculture, pisciculture and salt production [22,23].

The salt pans *(Mithache Agor or Mithagar)* in Goa experience three phases; namely the monsoons ceased phase, the salt pan preparatory phase and the salt harvesting phase [2]. These salt pans are located in close proximity to the sea or may be located on the estuaries of a river. During monsoons, salt pans lie submerged in rain water and therefore abandoned or utilized for aquaculture for breeding fishes, shrimps and prawns [24].

### a) Preparative phase (December to January)

The preparation of salt pans is carried out from December to January. The previous embankments (dykes/bunds) that were damaged due to the monsoon are repaired. Rain water / sea water from the salterns is drained using motor pumps. Once the water has been completely drained, the preparation of salt pan beds begins. The beds are ploughed, levelled by stamping and/ using a device called ‘*saalon*’. The extra clay is raked onto the walls of the bunds. *Saalon* has a long bamboo stick, approximately of 4 m in length, attached to a circular wooden base. During this process the borders of different pans are also constructed with clay.

The salterns consists of three distinct pans namely; reservoir pans (*tapovanim* / *tapounni*), evaporator pans (*podshing*) and crystallizer pans (*pikechem agor*) [25]. All the pans are inter-connected through an opening at the corners. Reservoir pan is used for receiving the sea water during tidal influxes and is connected to many evaporator pans. Crystallizer pans in turn is fed by the evaporator pans. The dimension of reservoir pan is 18–20 × 10–12 m, while that of the evaporator pan is of 18–20 × 6–8 m. In some salt pans the reservoir pan may be of the same size as evaporator pan; however the main difference is the depth of these two pans. Reservoir pans are around 20 in. deep, maintaining sea water level of upto 15–18 in. deep while the evaporator pans are 10 in. deep in which water is filled for upto 5 in. The reservoir pan is twice or thrice the size of the crystallizer pan. The dimension of crystallizer pan is 6.5–8 × 4–5 m and the depth is of same as that of the evaporator pan. The brine level in the crystallizer pan is maintained at a maximum level of 3 in. The size of evaporator pans plays a critical role in the production of salt. Bigger the size of evaporator pan, better the production of salt *(personal communication)*.

The reservoir pan is connected with the creek or canals, supplying seawater, during tidal influxes, through a sluice gate (*Manos*). Sluice is made of wood and the gates are made up of clay mixed with hay. This helps regulating the flow of water during the monsoon rains and tidal fluctuations. It helps in the controlled release of seawater into the reservoir pan during high tide and prevents the backflow of water during the low tide, thereby maintaining the level of water in the reservoir pan. Algal growth occurs in these pans which is harvested regularly and used as fertilizers for coconut and cashew plantations. The reservoir pans are also used for pisciculture especially for breeding salt water fishes, during the months of October to December [26].

**Figure 3:**
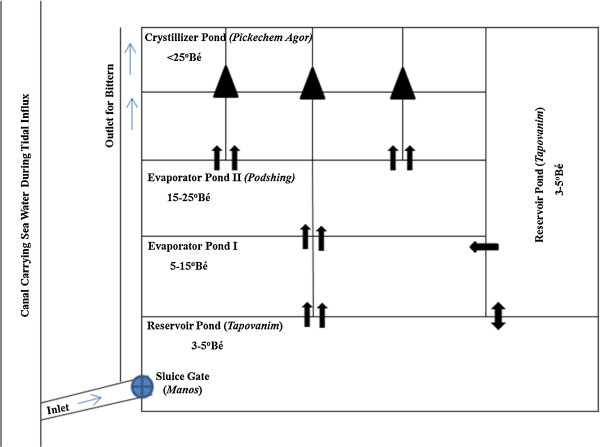
**Layout of a typical solar saltern of Goa.** →Indicates brine movement. ▲Crude salt crystal heaps.

Once the salinity of seawater in the reservoir pans is around 5ºBé, it is released to the first evaporator pan. Calcium carbonate (CaCO_3_) starts precipitating at a salinity around 5ºBé [27] in the reservoir pan and completely precipitates in the first evaporator pan. Once the brine attains salinity around 13–15ºBé, it is released from the first evaporator pan to the second evaporator pan. In the second evaporator pan, calcium sulphate (CaSO_4_) crystallizes in the form of gypsum. These precipitates form a hard crust at the bed of the evaporator pans. The brine, now having a salinity around 23–25ºBé, is released from the second evaporator pan to the crystallizer pan. Sodium chloride (NaCl) crystallizes around 27ºBé, first as flakes which float on the surface (*sai*) and then settle at the bottom of the pan (Figure 3). The brine in the crystallizer pan appears to be frothing due to the crystallisation of salt. The workers monitor the salinity of each pan by tasting the brine.

During the preparatory phase, seawater in the evaporator and crystallizer pan is allowed to stand and stirred time and again for about 20-25 days using a teeth shaped tool called ‘*danto*’. *Danto* has a long stick approximately 4 m in length, attached to a tool with teeth like projections [27]. The fed water is allowed to evaporate completely and the pans are fed again. This is done for removing the extra clay, which in turn will be raked onto the walls of the pan, thus further setting the salt pan beds. Most of the salterns will have a layout as described in Figure 3, however depending on the total area of the solar saltern, smaller salterns may not have separate evaporator pans.

Once the beds are set fresh brine is released from the reservoir to the evaporator pan and finally to the crystallizer pan for salt crystallization. It takes upto 10 days for the salt to crystallize during the first harvesting. Some crude salt (approximately 50–60 kg) is sprinkled over the crystallizer pans for aiding the salt crystallisation. This is repeated two to three times. When the salt crystallises, it is left on the pan unharvested for the initial three to four times. This is done for hardening the beds and making the beds uniform for further harvesting. The first salt harvested, contains lots of impurities mainly suspended clay particles and is brown to grey in colour [4,25].

### b) Salt harvesting phase (February to May)

When the salt pans are completely prepared, the peak salt harvesting season begins, usually from mid-February and lasts till end of May or early June, depending on the monsoons.

The brine in the evaporator pan having a salinity of 23–25 ºBé is released to the crystallizer pan every morning. The NaCl crystallizes out at around 27ºBé in the crystallizer pan and is harvested in the evening on a daily basis. The salt crystals are harvested with the wooden rake, *‘foyem’* (a long stick of approximately 4 m in length, attached to a wooden rectangular block of 50–70 × 15–20 cm) and piled as small heaps at the intersection of the pans [2]. In the Pernem taluka, the salt crystals are heaped at the centre of the bunds. If the brine is kept for a longer time, salts of magnesium and potassium will co-precipitate, making the salt bitter and unfit for consumption. In such situations, the entire brine is let off in the drain and the process restarted. Once sodium chloride precipitates out, the remaining brine rich in magnesium and potassium (bittern/mother liquor) is drained off in to the canals *(personal communication)*(Additional file 1: Figure S1).

The harvested salt is further washed with the concentrated brine solution to remove impurities if any and allowed to drain on the bunds itself. It is then transferred with bamboo baskets into a large heap off the salt pans before transferring it to a store house. The salt produced is transported to the local market through pickup vans.

The salt produced in Goa is of two grades: (i) Fertilizer grade and (ii) Preservative / Consumption grade. The salt harvested initially (for a month) will be of fertilizer grade because of the impurities of mud and clay contained. It is mostly used as manure or as soil conditioner and termite repellent for coconut trees. This salt is also used for the salting or preserving dry fish. The salt produced after the fine setting of the bed is the white consumption grade salt. This is used as a brine solution for pickling raw mangoes and in cuisine. The salt produced in different areas may render unique taste owing to the soil texture [25,26,28,29].

### Studies on biodiversity of solar salterns

Solar salterns are extreme environments that act as a niche for organisms which thrive over a range of extreme salinities, temperatures, pH, nutrient concentrations, oxygen availability, water activity and solar radiation. Microorganisms which survive in such high salinities are known as halophilic extremophiles, which include bacteria, archaea and fungi. Solar salterns are man-made ecosystems showing diversity of microbial populations during different stages of salt crystallisation [30-34]. These microorganisms play a vital role in the recycling of materials (nutrients and other substances) in saltern ecosystem and therefore, important members of biogeochemical cycles [35,36]. Since the estuarine regions are situated in the vicinity of thickly populated areas, they experience pollution from anthropogenic activities. Adding to this, barges carrying mineral ores pollute the rivers and estuarine regions with crude oil and heavy metals. As a consequence of this, the brine gets concentrated along with the metals [37-41]. Diversity studies on bacteria indicated the presence of members belonging to genera *Aeromonas*, *Pseudomonas*, *Vibrio*, *Desulfobacter, Desulfovibrio, Desulfococcus* and *Chromohalobacter*. These bacteria play an important role in the cycling of substances within the saltern ecosystem [35,36,42,43]. Culture dependent haloarchaeal diversity studies in the salterns of Goa indicated that *Halococcus sp.* are the dominant haloarchaeal member during less saline conditions. During salt harvesting phase, members belonging to the genera *Halococcus*, *Halorubrum*, *Haloarcula* and *Haloferax* have been identified [2]. Culture independent studies reported the presence of novel archaeal members belonging to phylum *Crenarchaeota* and *Euryarchaeota* in various salterns of Goa [44]. Fungal communities in salt pans of Goa were dominated by members belonging to the genera *Aspergillus* and *Penicillium* such as *Aspergillus versicolor, A. wentii, A. candidus, A. penicilloides A. flavus, A. sydowii, Penicillium chrysogenum, P. corylophilum, P. griseofulvum, Eurotium amstelodami* and *Hortaea werneckii*[45].

For any ecosystem to be successful, transport of energy across the food web should be regulated. Algae act as the sole producer in the salt pans producing energy by trapping sunlight. They provide food for crustaceans like *Artemia sp.*, for birds to feed on [46]. The dominant components of phytoplankton community in Goan salt pans were reported to be the members of *Cyanophyceae*, *Chlorophyceae*, *Bacillariophyceae* and *Dinophyceae*. The various species and their distribution showed strong correlation with the change in salinity. The salt pans contained algal members belonging to *Pediastrum* sp., *Oedogonium* sp., *Cladophora* sp., *Tetraselmis* sp., *Spirulina* sp. and *Spirogyra* sp. *Dichotomosiphon salina* and *Enteromorpha flexuosa*. Filamentous algae such as *Oscillatoria* sp. and *Phormidium* sp. and diatoms such as *Pleurosigma* sp., *Navicula* sp., *Chaetoceros* sp., *Amphora* sp., *Coscinodiscus* sp., *Surirella* sp. and *Nitzschia* sp. were common in all pans during less saline conditions but the growth of *Dunaliella* sp. were found to be dominating with the increase in salinity. More than 90% of the micro zooplankton in salt pans is dominated by ciliates viz., *Fabrea salina, Brachionus* sp.*, Tintinnids, Indomysis* sp. and *Artemia* sp. *F. salina* were observed between December and May but they were seen to be absent during the monsoon period. The microzooplankton diversity and species richness was found to be poor in the Goan salt pans [47-52]. The salt pans represent a specific ecosystem which depends not only on the salinity but also on the temperature. It permits the growth of only a few adapted organisms which can survive the extreme variations in the environmental conditions.

Since most of the salt pans of Goa lie in estuarine regions, the plants typical of mangrove vegetation dominate the surrounding areas [53]. Vegetation found surrounding the salt pans and bordering the creeks in rows of 4–8 are *Rhizophora mucronata* and *Avicennia officinalis*. Few trees of *Bruguiera gymnorrhiza*, *Sonneratia acida*, *Kandelia rheedei*, *Excoecaria agallocha* are also present along with shrubs of *Acanthus ilicifolius* and *Bruguiera parviflora*. Among these species *R. mucronata* and *S. acida* has shown tolerance to salinity till 30ºBé [54]. These plants provide additional reinforcements for the embankments by checking the floods during rainy season.

Salt pans don’t serve as nesting grounds for birds because of the continuous human disturbances. However a thick lush of mangroves provide shelter for many resident birds and migrating birds. However salt pans are excellent feeding grounds because of the availability of planktons like *Artemia sp.*[55]. Apart from the brine shrimps, fishes can also attract a large number of piscivorous birds. Birds like Indian Cormorant *Phalacrocorax fuscicollis*, Marsh sandpiper *Tringa stagnatilis*, Little Stint *Calidris minuta*, Jungle Myna *Acridotheres fuscus*, Plovers like *Charadrius dubius*, *Charadrius alexandrinus*, *Pluvialis fulva*, *Vanellus indicus*, Egrets like *Casmerodius albus*, *Egretta garzetta*, *Egretta gularis* and grey heron *Ardea cinerea* have been sighted regularly on the salt pans and they feed mostly on the crustaceans and fishes in and around the salt pans. Since, Goa is located in the Central Asian – Indian Flyway, the salt pans provide an attractive source of food for the migratory birds. Birds like pintail duck *Anas acuta*, northern shoveller *Anas clypeata*, storks like *Anastomus oscitans*, *Ciconia episcopus* and *Leptoptilos javanicus*, redshanks *Tringa tetanus*, sandpipers like *Actitis hypoleucos*, *Calidris ferruginea*, *Limicola falcinellus*, *Xenus cinereus* were found in the regions surrounding the salt pans in winters. Birds that were recorded in summer included brown-headed gulls *Larus brunnicephalus* and terns like *Gelochelidon nilotica*, *Sterna acuticauda* and *Chlidonias hybrida*[56,57]. Salt pans are shallow water bodies making it easy for birds to feed on the benthic communities and thereby regulating the growth of benthic invertebrates. Salt pans thus provide an excellent demonstration of energy flow through the food web.

## Conclusions

Salt production has played an irreplaceable role in the lives of Goan people. It is important to take measures for preventing the loss of these unique ecosystems. Apart from producing salt, it can be employed for pisciculture, aquaculture and agriculture. Awareness of the proper physicochemical and biological management of the salt pans can lead in the co-cultivation of algae like *Dunaliella sp.*, shrimps, etc. and could be used as natural fermenters for large scale cultivation of halophilic archaea, along with salt harvesting. Salt pans are good model for studying the ecological succession of organisms ranging from microbes to avifauna.

Salt making process is simple but extremely laborious. The workers working in these salt pans experience fatigue as they have to put in 8–12 h of work under the merciless sun, many times with a bending posture. The workers collect the salt with bare hands and walk about bare foot to maintain cleanliness. Although, saline water has been used as a therapy to ease arthritic pain, excess of exposure to salt leads to skin rashes. Reflection of the sun or UV rays off the salt also leads to a blinding effect [58]. The workers should be provided with gumboots, gloves and goggles to protect them from the high solar radiation. Simple machinery could be introduced for mechanised salt collection and piling.

Embankments should be constructed strong enough for withstanding the floods during rainy season and it should be checked at frequent time intervals for any leakages. The present government is now encouraging salt farming in traditional salt pans through the announcements of subsidies or financial support schemes [23]. Cooperative societies can be established for procuring the salt produced from the manufacturers and distributing it in the markets. The salt pans could also be encouraged for ecotourism. It is of utmost importance to protect the existing salt pans and thereby protect their contribution to the global ecological scenario.

## Competing interests

The authors declare that they have no competing interests.

## Authors’ contributions

All the authors conducted field visits to various saltpans, interviewed various salt pan workers and conducted extensive literature survey. All authors have written, read and approved the final manuscript.

## Supplementary Material

Additional file 1 **Figure S1.** Traditional salt harvesting during various stages of salt production (a-d) in Goa with the different tools (f and e) employed during the process and the use of the area for pisciculture (g). Click here for file
