# Supplementary material for: Community solar salt production in Goa, India
Source: Aquat Biosyst. 2012 Dec 1;8:30. doi: 10.1186/2046-9063-8-30 (PMC3543363; doi:10.1186/2046-9063-8-30)
Supplement: Additional file 1 — Figure S1. Traditional salt harvesting during various stages of salt production (a-d) in Goa with the different tools (f and e) employed during the process and the use of the area for pisciculture (g). [file 2046-9063-8-30-S1.docx]

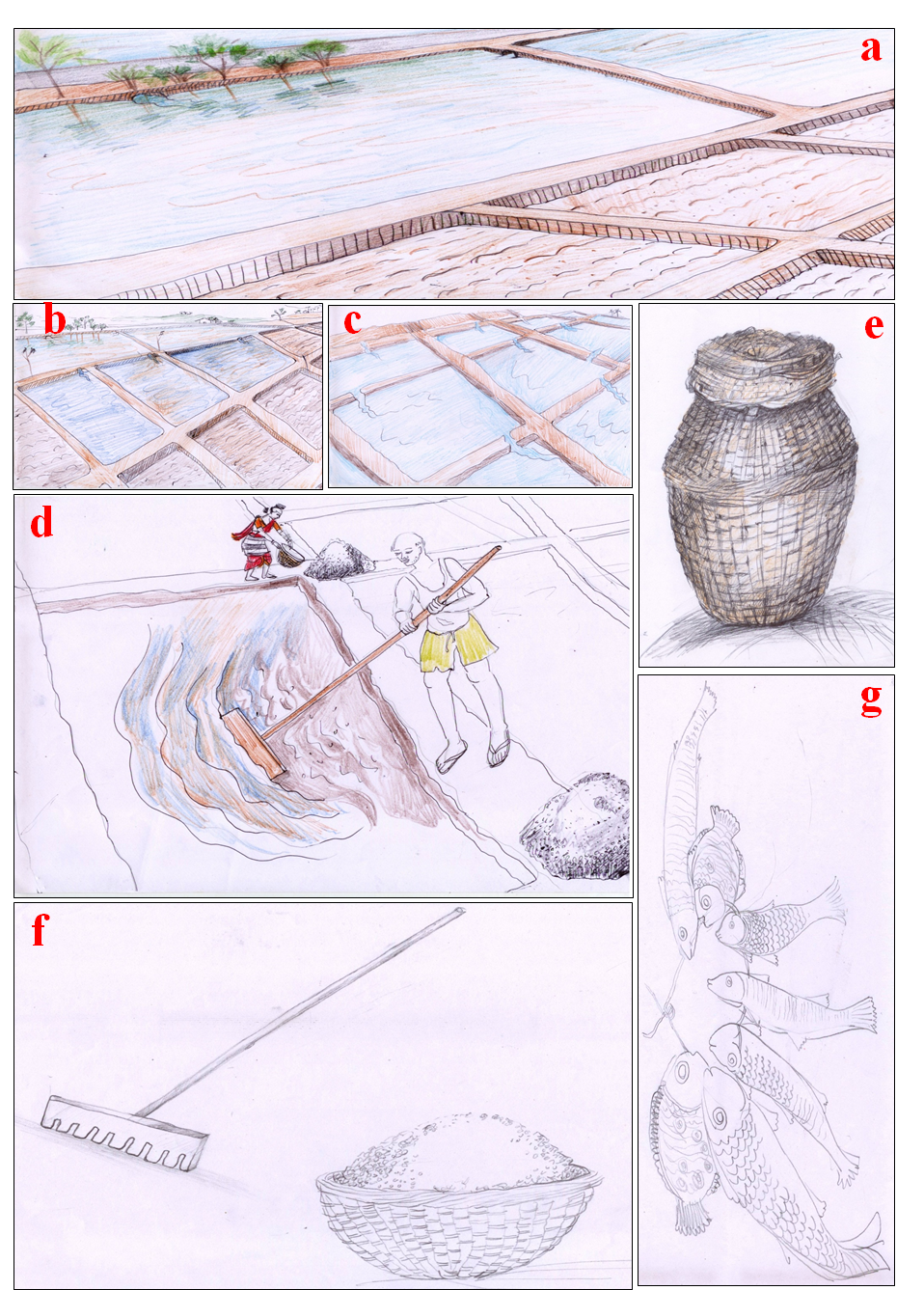


**Figure S1:** Traditional salt harvesting during various stages of salt production (a-d) in Goa with the different tools (f and e) employed during the process and the use of the area for pisciculture (g).

a) Entry of seawater into reservoir ponds which are at the periphery of salterns.

b) Movement of brine from the reservoir ponds to the evaporator ponds through channels of breaks in the dykes (*bunds*) connecting both ponds.

c) Entry of brine into the crystalliser ponds which immediately follow the evaporator ponds again by creating breaks in the dykes (*bunds*) connecting both ponds

d) A man is harvesting the salt using the *foyem*. A lady is collecting the precipitated salt using a container *pathlo.* The salt is heaped and kept for drying at the corners of the crystallizer ponds

e) A traditional container (*korond*) made of bamboo and coated with cow dung used for annual storage of salt by most of the natives of Goa.

f) A container (*pathlo*) made of bamboo used for carrying salt and a tool *danto* made of bamboo handle and teeth shaped base used for mixing of the brine in the crystallizer ponds.

g) Different fishes raised (pisciculture) in the saltpans during monsoons to winter (October to Feburary).
